# 3-(4-Bromo­phenyl­sulfon­yl)-5-ethyl-2-methyl-1-benzofuran

**DOI:** 10.1107/S1600536812048313

**Published:** 2012-11-30

**Authors:** Hong Dae Choi, Pil Ja Seo, Uk Lee

**Affiliations:** a Department of Chemistry, Dongeui University, San 24 Kaya-dong, Busanjin-gu, Busan 614-714, Republic of Korea; b Department of Chemistry, Pukyong National University, 599-1 Daeyeon 3-dong, Nam-gu, Busan 608-737, Republic of Korea

## Abstract

In the title compound, C_17_H_15_BrO_3_S, the 4-bromo­phenyl ring makes a dihedral angle of 76.58 (9)° with the mean plane [r.m.s. deviation = 0.006 (2) Å] of the benzofuran fragment. In the crystal, mol­ecules are linked by weak C—H⋯O and C—H⋯π inter­actions.

## Related literature

For the biological activity of benzofuran compounds, see: Aslam *et al.* (2009 ▶); Galal *et al.* (2009 ▶); Khan *et al.* (2005 ▶). For the crystal structures of related compounds, see: Choi *et al.* (2010 ▶, 2011 ▶).
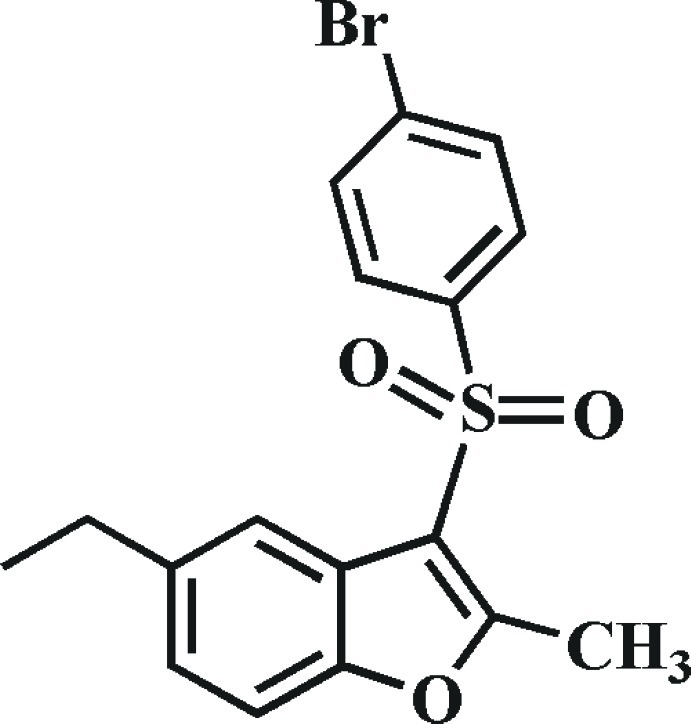


## Experimental

### 

#### Crystal data

C_17_H_15_BrO_3_S
*M* *_r_* = 379.26
Tetragonal, 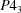

*a* = 10.2785 (3) Å
*c* = 15.2899 (6) Å
*V* = 1615.34 (9) Å^3^
*Z* = 4
Mo *K*α radiation
μ = 2.68 mm^−1^
*T* = 173 K
0.31 × 0.17 × 0.15 mm

#### Data collection

Bruker SMART APEXII CCD diffractometer
Absorption correction: multi-scan (*SADABS*; Bruker, 2009 ▶) *T*_min_ = 0.450, *T*_max_ = 0.746
8518 measured reflections
3085 independent reflections
2558 reflections with *I* > 2σ(*I*)
*R*_int_ = 0.035

#### Refinement

*R*[*F* ^2^ > 2σ(*F* ^2^)] = 0.034
*wR*(*F* ^2^) = 0.067
*S* = 1.03
3085 reflections
201 parameters
1 restraint
H-atom parameters constrained
Δρ_max_ = 0.35 e Å^−3^
Δρ_min_ = −0.29 e Å^−3^
Absolute structure: Flack (1983 ▶), 1000 Friedel pairs
Flack parameter: 0.001 (7)

### 

Data collection: *APEX2* (Bruker, 2009 ▶); cell refinement: *SAINT* (Bruker, 2009 ▶); data reduction: *SAINT*; program(s) used to solve structure: *SHELXS97* (Sheldrick, 2008 ▶); program(s) used to refine structure: *SHELXL97* (Sheldrick, 2008 ▶); molecular graphics: *ORTEP-3* (Farrugia, 2012 ▶) and *DIAMOND* (Brandenburg, 1998 ▶); software used to prepare material for publication: *SHELXL97*.

## Supplementary Material

Crystal structure: contains datablock(s) global, I. DOI: 10.1107/S1600536812048313/rn2111sup1.cif

Structure factors: contains datablock(s) I. DOI: 10.1107/S1600536812048313/rn2111Isup2.hkl

Supplementary material file. DOI: 10.1107/S1600536812048313/rn2111Isup3.cml

Additional supplementary materials:  crystallographic information; 3D view; checkCIF report

## Figures and Tables

**Table 1:** Hydrogen-bond geometry (Å, °) *Cg* is the centroid of the C2–C7 benzene ring.

*D*—H⋯*A*	*D*—H	H⋯*A*	*D*⋯*A*	*D*—H⋯*A*
C6—H6⋯O3^i^	0.95	2.53	3.238 (4)	131
C11—H11*A*⋯O3^ii^	0.98	2.58	3.321 (4)	132
C14—H14⋯*Cg* ^iii^	0.95	2.70	3.495 (4)	142

Symmetry codes: (i) 
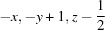
; (ii) 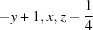; (iii) 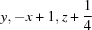.

**Fig. 1.**
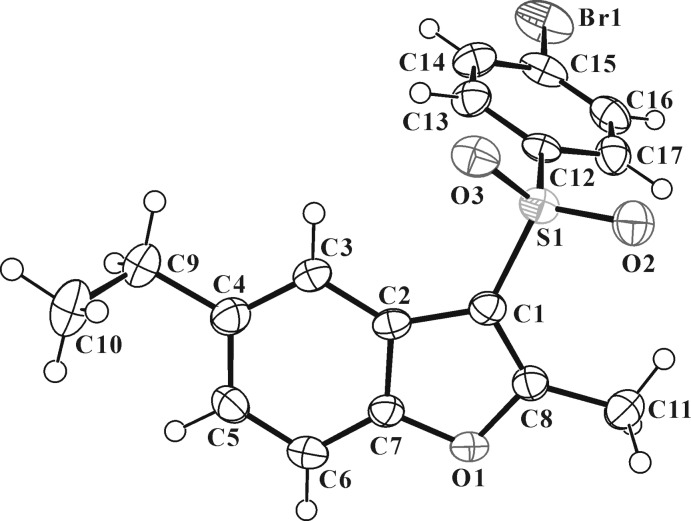
The molecular structure of the title compound with the atom numbering scheme. Displacement ellipsoids are drawn at the 50% probability level. H atoms are presented as small spheres of arbitrary radius.

**Fig. 2.**
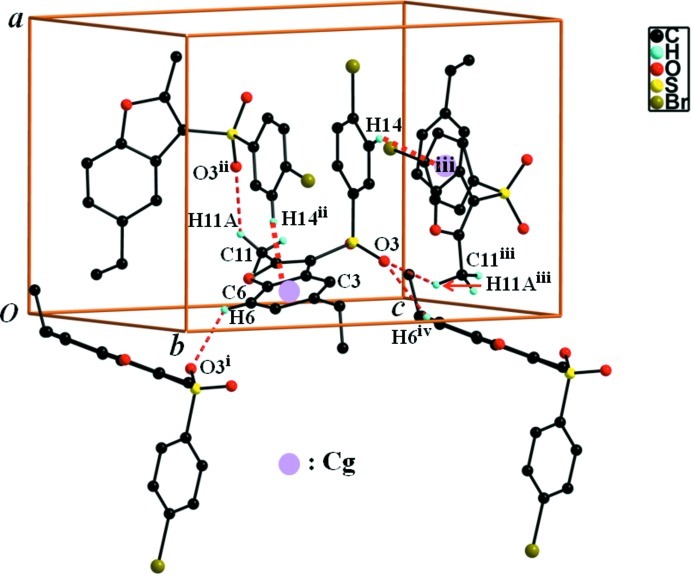
A view of the C—H···O and C—H···π interactions (dotted lines) in the crystal structure of the title compound. H atoms not participating in hydrogen-bonding were omitted for clarity. [Symmetry code: (i) - x, - y + 1, z - 1/2 (ii) - y + 1, x, z - 1/4 (iii) y, - x + 1, z + 1/4 (iv) - x, - y + 1, z + 1/2

## supplementary crystallographic information

### Comment

Many compounds containing the benzofuran skeleton have attracted much interest owing to their biological properties such as antibacterial and antifungal, antitumor and antiviral, and antimicrobial activities (Aslam *et al.*, 2009; Galal *et al.*, 2009; Khan *et al.*, 2005). As a part of our ongoing study of 5-ethyl-2-methyl-1-benzofuran derivatives containing 4-fluorophenylsulfonyl (Choi *et al.*, 2010) or 3-fluorophenylsulfonyl (Choi *et al.*, 2011) substituents in the 3-position, we report herein the crystal structure of the title compound.

The title compound crystallizes as the non-centrosymmetric space group *P*4_3_ in spite of having no asymmetric C atoms.

In the title molecule (Fig. 1), the benzofuran unit is essentially planar, with a mean deviation of 0.006 (2) Å from the least-squares plane defined by the nine constituent atoms. The dihedral angle between the 4-bromophenyl ring and the mean plane of the benzofuran ring is 76.58 (9)°. In the crystal structure (Fig. 2), molecules are connected by weak C—H···O and C—H···π interactions (Table 1, Cg is the centroid of the C2–C7 benzene ring).

### Experimental

3-Chloroperoxybenzoic acid (77%, 448 mg, 2.0 mmol) was added in small portions to a stirred solution of 3-(4-bromophenylsulfanyl)-5-ethyl-2-methyl-1-benzofuran (312 mg, 0.9 mmol) in dichloromethane (50 mL) at 273 K. After being stirred at room temperature for 10h, the mixture was washed with saturated sodium bicarbonate solution and the organic layer was separated, dried over magnesium sulfate, filtered and concentrated at reduced pressure. The residue was purified by column chromatography (benzene) to afford the title compound as a colorless solid [yield 68%, m.p. 404–405 K; *R*_f_ = 0.61 (benzene)]. Single crystals suitable for X-ray diffraction were prepared by slow evaporation of a solution of the title compound in diisopropyl ether at room temperature.

### Refinement

All H atoms were positioned geometrically and refined using a riding model, with C–H = 0.95 Å for aryl, 0.99 Å for methylene and 0.98 Å for methyl H atoms, respectively. *U*_iso_(H) = 1.2*U*_eq_(C) for aryl, methylene, and 1.5*U*_eq_(C) for methyl H atoms. The positions of methyl hydrogens were optimized rotationally.

### Crystal data

**Table:**

C_17_H_15_BrO_3_S	*D*_x_ = 1.559 Mg m^−^^3^
*M**_r_* = 379.26	Melting point = 404–405 K
Tetragonal, *P*4_3_	Mo *K*α radiation, λ = 0.71073 Å
Hall symbol: P 4cw	Cell parameters from 2851 reflections
*a* = 10.2785 (3) Å	θ = 2.4–23.1°
*c* = 15.2899 (6) Å	µ = 2.68 mm^−^^1^
*V* = 1615.34 (9) Å^3^	*T* = 173 K
*Z* = 4	Block, colourless
*F*(000) = 768	0.31 × 0.17 × 0.15 mm

### Data collection

**Table:**

Bruker SMART APEXII CCD diffractometer	3085 independent reflections
Radiation source: rotating anode	2558 reflections with *I* > 2σ(*I*)
Graphite multilayer monochromator	*R*_int_ = 0.035
Detector resolution: 10.0 pixels mm^-1^	θ_max_ = 28.3°, θ_min_ = 2.0°
φ and ω scans	*h* = −12→13
Absorption correction: multi-scan (*SADABS*; Bruker, 2009)	*k* = −13→12
*T*_min_ = 0.450, *T*_max_ = 0.746	*l* = −20→13
8518 measured reflections	

### Refinement

**Table:**

Refinement on *F*^2^	Secondary atom site location: difference Fourier map
Least-squares matrix: full	Hydrogen site location: difference Fourier map
*R*[*F*^2^ > 2σ(*F*^2^)] = 0.034	H-atom parameters constrained
*wR*(*F*^2^) = 0.067	*w* = 1/[σ^2^(*F*_o_^2^) + (0.0094*P*)^2^ + 0.1451*P*] where *P* = (*F*_o_^2^ + 2*F*_c_^2^)/3
*S* = 1.03	(Δ/σ)_max_ = 0.001
3085 reflections	Δρ_max_ = 0.35 e Å^−^^3^
201 parameters	Δρ_min_ = −0.29 e Å^−^^3^
1 restraint	Absolute structure: Flack (1983), 1000 Friedel pairs
Primary atom site location: structure-invariant direct methods	Flack parameter: 0.001 (7)

### Special details

**Table:**

Geometry. All esds (except the esd in the dihedral angle between two l.s. planes) are estimated using the full covariance matrix. The cell esds are taken into account individually in the estimation of esds in distances, angles and torsion angles; correlations between esds in cell parameters are only used when they are defined by crystal symmetry. An approximate (isotropic) treatment of cell esds is used for estimating esds involving l.s. planes.
Refinement. Refinement of F^2^ against ALL reflections. The weighted R-factor wR and goodness of fit S are based on F^2^, conventional R-factors R are based on F, with F set to zero for negative F^2^. The threshold expression of F^2^ > 2sigma(F^2^) is used only for calculating R-factors(gt) etc. and is not relevant to the choice of reflections for refinement. R-factors based on F^2^ are statistically about twice as large as those based on F, and R- factors based on ALL data will be even larger.

### Fractional atomic coordinates and isotropic or equivalent isotropic displacement parameters (Å^2^)

**Table:**

	*x*	*y*	*z*	*U*_iso_*/*U*_eq_	
Br1	0.83341 (3)	0.52123 (5)	0.64403 (3)	0.06379 (15)	
S1	0.22891 (7)	0.40370 (7)	0.69506 (5)	0.02917 (17)	
O1	0.1117 (2)	0.2989 (2)	0.46167 (13)	0.0331 (5)	
O2	0.2142 (2)	0.2825 (2)	0.73957 (15)	0.0401 (6)	
O3	0.1697 (2)	0.5188 (2)	0.73023 (15)	0.0372 (5)	
C12	0.3955 (3)	0.4357 (3)	0.68213 (18)	0.0248 (6)	
C2	0.1292 (3)	0.4919 (3)	0.5329 (2)	0.0250 (6)	
C17	0.4835 (3)	0.3333 (3)	0.6829 (2)	0.0357 (7)	
H17	0.4546	0.2464	0.6912	0.043*	
C13	0.4374 (3)	0.5626 (3)	0.6707 (2)	0.0346 (8)	
H13	0.3763	0.6319	0.6705	0.042*	
C16	0.6140 (3)	0.3604 (3)	0.6713 (2)	0.0394 (8)	
H16	0.6757	0.2916	0.6712	0.047*	
C15	0.6542 (3)	0.4857 (4)	0.66010 (19)	0.0390 (8)	
C6	0.0458 (3)	0.4963 (3)	0.3844 (2)	0.0370 (7)	
H6	0.0223	0.4525	0.3320	0.044*	
C7	0.0933 (3)	0.4311 (3)	0.4558 (2)	0.0287 (7)	
C5	0.0339 (3)	0.6299 (3)	0.3931 (3)	0.0382 (7)	
H5	0.0008	0.6787	0.3452	0.046*	
C3	0.1165 (3)	0.6262 (3)	0.5399 (2)	0.0299 (7)	
H3	0.1403	0.6696	0.5923	0.036*	
C8	0.1590 (3)	0.2747 (3)	0.5441 (2)	0.0295 (7)	
C9	0.0514 (4)	0.8410 (3)	0.4760 (3)	0.0454 (9)	
H9A	0.0846	0.8705	0.5335	0.054*	
H9B	0.1046	0.8832	0.4301	0.054*	
C14	0.5674 (3)	0.5881 (3)	0.6595 (2)	0.0392 (8)	
H14	0.5971	0.6748	0.6515	0.047*	
C4	0.0687 (3)	0.6956 (3)	0.4694 (2)	0.0359 (8)	
C1	0.1709 (3)	0.3870 (3)	0.58850 (19)	0.0275 (7)	
C11	0.1847 (3)	0.1373 (3)	0.5637 (2)	0.0399 (8)	
H11A	0.2469	0.1025	0.5212	0.060*	
H11B	0.1033	0.0880	0.5603	0.060*	
H11C	0.2210	0.1297	0.6228	0.060*	
C10	−0.0874 (4)	0.8851 (4)	0.4663 (3)	0.0598 (11)	
H10A	−0.1202	0.8595	0.4086	0.090*	
H10B	−0.0917	0.9799	0.4721	0.090*	
H10C	−0.1409	0.8446	0.5119	0.090*	

### Atomic displacement parameters (Å^2^)

**Table:**

	*U*^11^	*U*^22^	*U*^33^	*U*^12^	*U*^13^	*U*^23^
Br1	0.02640 (18)	0.1092 (4)	0.0558 (2)	−0.00760 (19)	0.00298 (19)	−0.0095 (3)
S1	0.0271 (4)	0.0358 (4)	0.0246 (3)	−0.0022 (3)	0.0028 (3)	−0.0006 (4)
O1	0.0342 (12)	0.0308 (12)	0.0344 (12)	−0.0039 (9)	−0.0097 (10)	−0.0053 (10)
O2	0.0447 (14)	0.0415 (14)	0.0340 (12)	−0.0079 (10)	0.0037 (11)	0.0077 (11)
O3	0.0330 (13)	0.0478 (14)	0.0308 (12)	0.0037 (10)	0.0048 (10)	−0.0083 (11)
C12	0.0189 (14)	0.0371 (17)	0.0183 (13)	−0.0009 (12)	0.0012 (12)	−0.0052 (13)
C2	0.0176 (14)	0.0285 (16)	0.0288 (16)	−0.0047 (12)	0.0025 (13)	−0.0043 (13)
C17	0.0399 (19)	0.0355 (18)	0.0316 (16)	0.0026 (13)	0.0022 (15)	0.0029 (15)
C13	0.0315 (17)	0.0337 (18)	0.0387 (19)	0.0036 (13)	0.0008 (14)	−0.0074 (14)
C16	0.0329 (18)	0.052 (2)	0.0330 (18)	0.0134 (15)	−0.0038 (14)	−0.0043 (16)
C15	0.0219 (15)	0.071 (3)	0.0243 (17)	−0.0017 (15)	0.0011 (14)	−0.0056 (17)
C6	0.0384 (18)	0.0387 (17)	0.0338 (17)	−0.0044 (14)	−0.0098 (16)	−0.0033 (16)
C7	0.0233 (15)	0.0279 (16)	0.0348 (17)	−0.0020 (12)	−0.0040 (13)	−0.0018 (14)
C5	0.0345 (18)	0.0388 (18)	0.0414 (17)	−0.0023 (14)	−0.0121 (17)	0.006 (2)
C3	0.0296 (17)	0.0273 (17)	0.0329 (17)	−0.0052 (13)	−0.0018 (14)	−0.0028 (14)
C8	0.0272 (17)	0.0302 (17)	0.0312 (17)	−0.0024 (13)	−0.0016 (14)	0.0012 (14)
C9	0.058 (2)	0.0264 (18)	0.052 (2)	−0.0003 (16)	−0.0081 (19)	0.0021 (16)
C14	0.0355 (18)	0.0398 (19)	0.042 (2)	−0.0093 (14)	0.0031 (16)	−0.0073 (16)
C4	0.0341 (18)	0.0313 (18)	0.0423 (19)	−0.0045 (13)	−0.0024 (15)	−0.0010 (15)
C1	0.0221 (16)	0.0302 (17)	0.0302 (16)	−0.0048 (12)	0.0000 (13)	0.0004 (14)
C11	0.043 (2)	0.0305 (18)	0.047 (2)	−0.0022 (15)	−0.0034 (17)	−0.0018 (16)
C10	0.072 (3)	0.034 (2)	0.074 (3)	0.0137 (18)	−0.004 (2)	0.000 (2)

### Geometric parameters (Å, º)

**Table:**

Br1—C15	1.894 (3)	C6—C5	1.384 (4)
S1—O2	1.427 (2)	C6—H6	0.9500
S1—O3	1.435 (2)	C5—C4	1.395 (5)
S1—C1	1.743 (3)	C5—H5	0.9500
S1—C12	1.754 (3)	C3—C4	1.382 (4)
O1—C8	1.374 (4)	C3—H3	0.9500
O1—C7	1.376 (3)	C8—C1	1.345 (4)
C12—C13	1.385 (4)	C8—C11	1.467 (4)
C12—C17	1.388 (4)	C9—C10	1.505 (5)
C2—C7	1.384 (4)	C9—C4	1.509 (4)
C2—C3	1.391 (4)	C9—H9A	0.9900
C2—C1	1.438 (4)	C9—H9B	0.9900
C17—C16	1.381 (4)	C14—H14	0.9500
C17—H17	0.9500	C11—H11A	0.9800
C13—C14	1.372 (4)	C11—H11B	0.9800
C13—H13	0.9500	C11—H11C	0.9800
C16—C15	1.364 (5)	C10—H10A	0.9800
C16—H16	0.9500	C10—H10B	0.9800
C15—C14	1.381 (5)	C10—H10C	0.9800
C6—C7	1.371 (4)		
			
O2—S1—O3	119.73 (14)	C4—C3—C2	119.0 (3)
O2—S1—C1	108.87 (14)	C4—C3—H3	120.5
O3—S1—C1	106.63 (14)	C2—C3—H3	120.5
O2—S1—C12	108.70 (14)	C1—C8—O1	109.9 (3)
O3—S1—C12	107.56 (13)	C1—C8—C11	134.9 (3)
C1—S1—C12	104.29 (13)	O1—C8—C11	115.2 (3)
C8—O1—C7	106.7 (2)	C10—C9—C4	113.8 (3)
C13—C12—C17	120.8 (3)	C10—C9—H9A	108.8
C13—C12—S1	119.7 (2)	C4—C9—H9A	108.8
C17—C12—S1	119.6 (2)	C10—C9—H9B	108.8
C7—C2—C3	119.2 (3)	C4—C9—H9B	108.8
C7—C2—C1	104.2 (2)	H9A—C9—H9B	107.7
C3—C2—C1	136.6 (3)	C13—C14—C15	118.9 (3)
C16—C17—C12	118.7 (3)	C13—C14—H14	120.6
C16—C17—H17	120.7	C15—C14—H14	120.6
C12—C17—H17	120.7	C3—C4—C5	119.6 (3)
C14—C13—C12	120.0 (3)	C3—C4—C9	120.1 (3)
C14—C13—H13	120.0	C5—C4—C9	120.3 (3)
C12—C13—H13	120.0	C8—C1—C2	108.5 (3)
C15—C16—C17	120.1 (3)	C8—C1—S1	126.0 (2)
C15—C16—H16	120.0	C2—C1—S1	125.5 (2)
C17—C16—H16	120.0	C8—C11—H11A	109.5
C16—C15—C14	121.7 (3)	C8—C11—H11B	109.5
C16—C15—Br1	119.6 (3)	H11A—C11—H11B	109.5
C14—C15—Br1	118.7 (3)	C8—C11—H11C	109.5
C7—C6—C5	116.1 (3)	H11A—C11—H11C	109.5
C7—C6—H6	122.0	H11B—C11—H11C	109.5
C5—C6—H6	122.0	C9—C10—H10A	109.5
C6—C7—O1	125.7 (3)	C9—C10—H10B	109.5
C6—C7—C2	123.6 (3)	H10A—C10—H10B	109.5
O1—C7—C2	110.7 (3)	C9—C10—H10C	109.5
C6—C5—C4	122.5 (3)	H10A—C10—H10C	109.5
C6—C5—H5	118.7	H10B—C10—H10C	109.5
C4—C5—H5	118.7		
			
O2—S1—C12—C13	−156.8 (2)	C7—O1—C8—C11	179.7 (3)
O3—S1—C12—C13	−25.8 (3)	C12—C13—C14—C15	−0.1 (5)
C1—S1—C12—C13	87.2 (3)	C16—C15—C14—C13	0.1 (5)
O2—S1—C12—C17	23.7 (3)	Br1—C15—C14—C13	179.6 (2)
O3—S1—C12—C17	154.7 (2)	C2—C3—C4—C5	0.2 (5)
C1—S1—C12—C17	−92.3 (3)	C2—C3—C4—C9	178.8 (3)
C13—C12—C17—C16	−0.5 (4)	C6—C5—C4—C3	−0.3 (5)
S1—C12—C17—C16	179.1 (2)	C6—C5—C4—C9	−178.8 (3)
C17—C12—C13—C14	0.3 (4)	C10—C9—C4—C3	−117.6 (4)
S1—C12—C13—C14	−179.3 (3)	C10—C9—C4—C5	60.9 (5)
C12—C17—C16—C15	0.5 (5)	O1—C8—C1—C2	0.1 (3)
C17—C16—C15—C14	−0.3 (5)	C11—C8—C1—C2	180.0 (3)
C17—C16—C15—Br1	−179.8 (2)	O1—C8—C1—S1	−179.2 (2)
C5—C6—C7—O1	179.4 (3)	C11—C8—C1—S1	0.7 (5)
C5—C6—C7—C2	−0.4 (5)	C7—C2—C1—C8	0.2 (3)
C8—O1—C7—C6	−179.2 (3)	C3—C2—C1—C8	178.9 (3)
C8—O1—C7—C2	0.6 (3)	C7—C2—C1—S1	179.5 (2)
C3—C2—C7—C6	0.4 (5)	C3—C2—C1—S1	−1.8 (5)
C1—C2—C7—C6	179.3 (3)	O2—S1—C1—C8	−22.4 (3)
C3—C2—C7—O1	−179.4 (3)	O3—S1—C1—C8	−152.8 (3)
C1—C2—C7—O1	−0.5 (3)	C12—S1—C1—C8	93.5 (3)
C7—C6—C5—C4	0.4 (5)	O2—S1—C1—C2	158.4 (2)
C7—C2—C3—C4	−0.2 (4)	O3—S1—C1—C2	28.0 (3)
C1—C2—C3—C4	−178.8 (3)	C12—S1—C1—C2	−85.7 (3)
C7—O1—C8—C1	−0.4 (3)		

### Hydrogen-bond geometry (Å, º)

Cg is the centroid of the C2–C7 benzene ring.

**Table:**

*D*—H···*A*	*D*—H	H···*A*	*D*···*A*	*D*—H···*A*
C6—H6···O3^i^	0.95	2.53	3.238 (4)	131
C11—H11*A*···O3^ii^	0.98	2.58	3.321 (4)	132
C14—H14···*Cg*^iii^	0.95	2.70	3.495 (4)	142

Symmetry codes: (i) −*x*, −*y*+1, *z*−1/2; (ii) −*y*+1, *x*, *z*−1/4; (iii) *y*, −*x*+1, *z*+1/4.
